# A pilot study to evaluate the efficacy of adding a structured home visiting intervention to improve outcomes for high-risk families attending the Incredible Years Parent Programme: study protocol for a randomised controlled trial

**DOI:** 10.1186/1745-6215-15-66

**Published:** 2014-02-25

**Authors:** Dianne G Lees, David M Fergusson, Christopher M Frampton, Sally N Merry

**Affiliations:** 1 Bay of Plenty District Health Board, Private Bag 12024, Tauranga 3141, New Zealand; 2 Otago University, Christchurch, P.O. Box 4345, Christchurch 8140, New Zealand; 3 University of Auckland, Private Bag 92019, Auckland 1141, New Zealand

**Keywords:** Conduct problems, Early childhood, Home coaching, Incredible Years Specialist Service, Randomised controlled trial

## Abstract

**Background:**

Antisocial behaviour and adult criminality often have their origins in childhood and are best addressed early in the child’s life using evidence-based treatments such as the ‘Incredible Years Parent Programme’. However, families with additional risk factors who are at highest risk for poor outcomes do not always make sufficient change while attending such programmes. Additional support to address barriers and improve implementation of positive parenting strategies while these families attend the Incredible Years Programme may improve overall outcomes.

The study aims to evaluate the efficacy of adding a structured home visiting intervention (Home Parent Support) to improve outcomes in families most at risk of poor treatment response from the Incredible Years intervention. This study will inform the design of a larger prospective randomised controlled trial.

**Methods/design:**

A pilot single-blind, parallel, superiority, randomised controlled trial. Randomisation will be undertaken using a computer-generated sequence in a 1:1 ratio to the two treatments arranged in permuted blocks with stratification by age, sex, and ethnicity. One hundred and twenty six participants enrolled in the Incredible Years Parent Programme who meet the high-risk criteria will be randomly allocated to receive either Incredible Years Parent Programme and Home Parent Support, or the Incredible Years Parent Programme alone. The Home Parent Support is a 10-session structured home visiting intervention provided by a trained therapist, alongside the usual Incredible Years Parent Programme, to enhance the adoption of key parenting skills. The primary outcome is the change in child behaviour from baseline to post-intervention in parent reported Eyberg Child Behavior Inventory Problem Scale.

**Discussion:**

This is the first formal evaluation of adding Home Parent Support alongside Incredible Years Parent Programme for families with risk factors who typically have poorer treatment outcomes. We anticipate that the intervention will help vulnerable families stay engaged, strengthen the adoption of effective parenting strategies, and improve outcomes for both the children and families.

**Trial registration:**

Australian New Zealand Clinical Trials Registry ACTRN12612000878875.

## Background

Child conduct problems are prevalent affecting an estimated 5 to 10% of children in New Zealand [1,2]. They negatively impact on parental wellbeing and result in increased demands on health, education, and social services [1,3]. Longitudinal studies have established that conduct problems in childhood are precursors to a range of adverse outcomes in adulthood [4,5]. Without effective intervention, these problems have the potential to lead to long-term problems including substance abuse, mental health difficulties, violent behaviour, and poor physical health [6]. Conduct problems, aggressive behaviour, and poor emotional regulation in young children are important predictors of later antisocial and criminal behaviour in some adolescents, and the effectiveness of interventions diminishes with age [1,2,4,6,7]. Therefore, it is prudent to identify those young people at risk and provide an evidence-based intervention early in the life of the child before problematic behaviours have become entrenched and parent–child relationships have broken down.

Intervening early in the life of the child has proven long-term benefits for children with challenging behaviour [5,8], and better outcomes for the family and the community than treatment in adolescent years [8,9]. Heckman [10] has identified the wider benefits from early childhood intervention, including improved learning in schools, as well as reduced crime, teenage pregnancy, and welfare dependency. Early childhood intervention is also cost-effective [3,11]. For example, Scott and colleagues [3] estimate the cost of public services used by an individual with conduct disorder to be ten times greater than an individual with no problems. Church [1] found similar costs in New Zealand with successful intervention for a 5-year-old costing approximately $5,000 compared to $60,000 for an adolescent. Furthermore, Church found the success rate is 70% greater for younger children.

Although genetic factors have a role in the development of challenging behaviour, it is the environmental factors that are more readily addressed. Behavioural and social learning theories posit that children learn behaviour within the context of their environment. Children raised in a positive and nurturing environment are more likely to have pro-social friendship skills, an ability to regulate their emotional responses, and achieve appropriate educational standards. On the other hand, children raised in environments with limited resources, by parents who have health problems, and who use punitive parenting practices are less likely to achieve good outcomes [2]. Intervening with an effective parenting programme has been shown to address many of the environmental factors contributing to the development of anti-social and aggressive behaviours in children, and improve their long-term outcomes [5,8].

The Incredible Years Parent Programme (IYP) is one evidence-based intervention with extensive research showing effectiveness for children with conduct problems [3,5,8,12,13]. Results have been replicated in a number of countries, e.g., Wales, Ireland, Norway, USA, Canada, England [14-17], and also for foster families where the children have additional high needs [18,19]. There is a small but growing body of literature demonstrating the effectiveness of IYP programmes in New Zealand, for example with Maori participants [9,20-22], single parents with children with Attention Deficit Hyperactivity Disorder [23], and within the Ministry of Education [22,24,25]. However, despite these good results, a third of children with behavioural problems whose parents attend IYP still experience difficulties and are at risk of developing chronic problems in adolescence [17,26]. In a trial with children initially within the clinical range, Webster-Stratton et al. [5] found that post-treatment child behaviour scores remaining within the clinical range was a predictor of adolescent engagement in delinquent acts; achieving post-treatment scores within the normal range was more likely to result in better long-term outcomes.

Those who do poorly despite treatment often have risk factors that are identifiable prior to intervention. While the literature is varied on which specific factors attribute to poor treatment outcomes, the factors generally cluster into four categories [7,15,27-30]:

i. Child variables (severity of child behaviour, referral source, sex).

ii. Parent variables (maternal psychopathology/depression, coercive/punitive parenting style, maternal age, negative life events/stressors).

iii. Family demographics (single parent, family size, low income, education/occupation, maternal age, minority status).

iv. Participation variables (treatment attendance, perceived barriers to treatment participation).

Other factors for poor response to treatment identified in the literature [12,15,28,31] and those observed from personal experience of delivering the programme (Unpublished) include lack of partner support, resistance to change in the home, parents’ unrealistic and developmentally inappropriate expectations for children, adverse child rearing practices, and negative cognitions and perceptions of child behaviour. Reyno and McGrath [29] concluded from their meta-analysis that providing additional support to parents attending parent training may improve outcomes for high risk families.

In recognition of the need to provide additional support for families attending the IYP programme, the New Zealand Ministries of Health and Education established the Incredible Years Specialist Service (IYSS) as a collaborative venture. This service provides a targeted intervention, Home Parent Support (HPS), for families of young children who are identified as greater risk of non-response to treatment for conduct problem behaviours. The specification for IYSS is to provide a comprehensive inter-agency intervention to address conduct/antisocial behaviour and associated mental health problems in children. Key features include:

• Strengthening and supporting a coordinated interagency response;

• Bringing mental health expertise and capacity to a multi-agency team;

• Strengthening interventions for Maori families;

• A focus on children aged 3–7 years; and

• Prioritising those with more severe conduct problems.

The joint commitment from the Health and Education sectors to work collaboratively should improve access to parent information, child health, and educational services for vulnerable families at an optimum time in the life of the child. It is expected that this support will improve engagement in IYP and improve overall outcomes. However, we do not have robust evidence that HPS does improve outcomes compared with IYP alone. The aim of this study is to evaluate the effectiveness of adding a structured home intervention while the parent/carer attends IYP.

As in all group parent programmes, most home visiting programmes are based on the premise that parents play an important role in shaping the outcomes of their children, and that intervention in early childhood ensures input in a sensitive developmental period [27,32]. There is also an increasing awareness of the importance of the early caregiving environment and the impact this has on early neurological development [33]. Over the last 20 years, there has been an increase in home visiting programmes in an attempt to address child maltreatment, reduce infant mortality, and improve child wellbeing [34]. Home visiting allows interventions to be tailored to the specific needs of the family and provides therapists with the opportunity to assess and address other risk factors such as substance abuse, poor parental mental health, and violence in the home [35].

In spite of the growing popularity of home visiting programmes, reviews report mixed results [33,35]. There are only a few programmes that have demonstrated long-term benefits for parents and children [36-38]. The diverse results of home visiting programmes, in general, give some indication of how difficult it is to change parenting practices once dysfunctional patterns have become the established norm for the family [34]. Gomby [35] suggests that combining an effective home visiting programme with other education programmes may improve outcomes.

Characteristics that contribute to an effective home visiting programme include internal consistency (adherence to the curriculum), a collaborative approach when working with parents, well trained and well supervised therapists, close relationship with other services, and low caseloads [33,35]. These factors are key components of the HPS intervention developed by DL to support families to maximise the benefits of IYP.

HPS provides a structured intervention for parents in their home in conjunction with attending IYP. Parents have the benefit of the IYP group curriculum on child development and parenting practices, experiential group learning, and socialisation. HPS is provided by therapists who are trained mental health workers and accredited IYP facilitators. They are familiar with the detail of the course content and key principles, and work collaboratively with the parents in their home. They support parents to implement the key parenting principles and practice new skills, and tailor these strategies to their own circumstances. Therapists focus on building the parent–child relationship and on addressing negative cognitions and coercive patterns of interaction. They also assess barriers for change and support parents to access other appropriate health and education services such as adult mental health services, income support, relationship services, and special education services. Therapists follow a structured guide to ensure adherence to the curriculum and they attend weekly supervision to maintain fidelity. In an open trial of HPS participants reported high levels of satisfaction and retention rate was high at 92% (Unpublished).

We hypothesise that the addition of a structured home intervention (HPS) will result in better outcomes for families with additional risk factors for poor treatment response, and we expect to increase the percentage of children with post-treatment scores in the non-clinical range. The current study has been designed to evaluate this intervention and, if it is found to be effective, there is the potential for national implementation.

The successful widespread implementation of any intervention requires a degree of pragmatism. To identify families at greater risk of non-response it would be unrealistic to try and screen for all the factors outlined above, and a number of them cluster together. Three domains have been used in this study to identify families at greater risk of non-response. These are from the categories of the overall risk factors and would be easy to implement in a community real-world setting.

1. Child behaviour scores in the clinical range on standard child behaviour psychometrics and/or school exclusion.

2. Poor parental mental health.

3. Involvement of Child Youth and Family Social Services indicting abuse or neglect (as a proxy measure of coercive parenting).

## Methods/design

### Design

This study is a pilot single-blind, parallel, superiority, randomised controlled trial. Eligible participants will be randomly allocated to receive IYP plus HPS or to the control group of IYP treatment alone. Randomisation will be undertaken using a computer-generated sequence in a 1:1 ratio to the two treatments arranged in permuted blocks. Stratification will be by age, sex, and ethnicity. Data from all participants will be included in the data analysis, irrespective of whether follow-up data is available using an intention-to-treat design.

### Ethical approval

Approval has been received from the New Zealand Northern B Health and Disability Ethics Committee (NTY/12/06/050).

### Setting

This study is being carried out in a real-world setting within the Bay of Plenty District Health Board, Tauranga, New Zealand.

### Participants

Participants are parents/caregivers of children with conduct problems recruited from IYP groups delivered in the community by the Child and Adolescent Mental Health Services (CAMHS), the Ministry of Education, and non-government organisations in Tauranga. Parents attending IYP are either self-referred or referred by health or education services. Criteria for parents to attend IYP are: they speak English, have the child in their custody or have regular access arrangements, and their child does not have an intellectual disability. All families attending IYP are screened for eligibility for IYSS and those who meet the criteria will be invited to take part in the trial until 126 participants have been recruited. Participants will be randomly allocated to IYP plus HPS or to IYP alone. Where there is more than one child in a family who meets the criteria for IYSS, the parent will identify the child they find most challenging as the focus child. Where more than one parent/carer is attending IYP, and their child meets the criteria for IYSS, one parent/carer will be identified as the trial participant.

### Inclusion criteria

Participants will be eligible for inclusion in the trial if:

• They are parents/caregivers of children with conduct problems, who are enrolled to attend IYP.

• Their child is over 3 years and under 8 years of age on the date of signed consent to participate in the trial.

• Parent child behaviour scores are in the clinical range for any of the following psychometrics: Eyberg Child Behavior Inventory (ECBI) total problem scale >11; ECBI intensity scale >127; Social Competency Scale (SCS) <17.

• Or there is one of the following risk factors: Child, Youth and Family Services involvement; school exclusion; parent diagnosed with mental health disorder.

### Exclusion criteria

None.

### Withdrawal criteria

Participants can withdraw from the intervention at any time but will remain in the trial. If participants require on-going support they will be assisted to engage in an appropriate community agency.

### Intervention

#### HPS

Participants will receive 10 in-home sessions from a separate therapist accredited in IYP whilst they attend the 14 to 16 week Basic IYP. The intervention will include a comprehensive child assessment, including developmental, medical, and social history, pre-school or school reports, involvement of other agencies, family structure, and parental mental health. Participants will be supported to identify specific goals they wish to achieve and record them. The therapist will visit them in their homes to provide support to personalise and implement the IYP strategies and to address any barriers to implementation of these strategies that they or the therapist identifies. The therapist will follow a structured intervention guide to ensure therapist fidelity. Treatment includes follow-up contact at one-month post-intervention to assess stability of change and provide further assistance if required.

The therapists delivering HPS will meet weekly to review all participants’ progress and identify any additional support required for families. Therapists will have fortnightly contact with IYP group leaders to review attendance and participant progress. Participants will be reviewed monthly by a multidisciplinary team that includes a Child and Adolescent Psychiatrist, Paediatrician, Ministry of Education IYP co-ordinator, and the HPS therapists. Specialist psychiatric and/or paediatric assessment is available if required. This multidisciplinary team will also review any adverse events and assess the likelihood that this may be related to the intervention.

#### Therapist guide

The therapist guide specifies the important components of the home intervention. It identifies key elements for each session to ensure the intervention is focussed on the content and learning from IYP and that the learning occurs in a supportive collaborative manner to encourage and motivate participants. The key elements of HPS include reviewing IYP principles, tailoring strategies, practicing and rehearsing new skills, therapist modelling praise and affirmation, identifying and reviewing participant goals, and addressing barriers to implementation of new skills.

#### Intervention fidelity

HPS therapists will follow the structured guide in their intervention and keep a record of activities in each session to ensure that key activities are included. This record will be reviewed in weekly supervision.

### Control

Participants will be in the same IYP groups as those in the intervention arm. This is to prevent real or perceived difference between the groups. All IYP groups will be delivered by trained facilitators in CAMHS, the Ministry of Education, or non-government organisations and will receive 2 hours supervision fortnightly. Those in the control will receive the usual support from IYP group leaders and will still have access to all services that would normally be available to them.

### Outcomes

#### Primary outcome

The primary outcome is a change in child behaviour from baseline to post-intervention in the parent-reported ECBI Problem scale.

#### Secondary outcomes

• The percentage of parent scores on the ECBI that are in the normal range at post-treatment.

• The percentage of parent scores on the Child SCS that are in the normal range at post-treatment.

• Changes from pre- to post-intervention in child behaviour, parenting practices, parent relationships, and parental well-being measured on the Family Questionnaire (FQ) scales.

• The percentage with at least 80% engagement in IYP measured on the attendance register.

• Levels of parent satisfaction with IYP measured using the Parent Satisfaction Questionnaire.

• Maintenance of improvement at 6-month follow-up measured on the FQ, ECBI, and SCS.

• Parent reports of competence with implementing IYP strategies in the home as reported in the Follow-up Questionnaire at 6 months.

### Measurements

#### Screening measurement

The IYP group leaders will carry out screening using the ECBI and the SCS – Parent Version. These measures have been used in similar studies [13,39].

• The ECBI is a parent-rated inventory with two scales. The total problem scale is a measure of the type and frequency of 36 behaviours. Total problem scores over 11 are in the clinical range. The intensity scale is the degree to which parents find the behaviours problematic, rated 1 to 7. Intensity scores over 127 are in the clinical range [40].

• The SCS – Parent Version was developed by the Conduct Problem Prevention Research Group [41,42]. It consists of 12 items completed by the parent on their child’s pro-social behaviours, communication skills, and self-control on a 5-point Likert scale. A total score less than 17 is indicative of poor social skills and is considered a clinically important cut-point for meeting IYSS criteria.

#### Baseline

Once eligibility is confirmed a research assistant will collect pre-intervention baseline data on demographics and the FQ. The FQ was developed by the Incredible Years Pilot Study Working Group for use in a joint-agency national evaluation of Incredible Years Pilot Study [43]. The questionnaire is a comprehensive assessment of child behaviour, parenting practices, partner relationships, parental depression, life events, cultural participation, and parent satisfaction. The research assistant will read all questions out to the participant and score responses on the questionnaire.

#### Post-treatment

The IYP group leaders will collect post-treatment measurements using the ECBI, SCS, and the standard Incredible Years Parent Satisfaction Questionnaire. This is a 24-question assessment of parent views on the programme content and teaching methods. Parents rate their satisfaction on 1- to 7-point Likert scale [44]. The research assistant will repeat the relevant sections of the FQ within two weeks of the final IYP session.

#### Follow-up

At the 6-month follow-up, the research assistant will collect ECBI, SCS, and FQ and a quantitative/qualitative follow-up questionnaire. This questionnaire includes Likert-type scales and opportunities for written feedback to assess levels of engagement, helpful aspects of the trial, level of competency with implementing IYP strategies, and changes in relationships and behaviour noticed by parent/carers (Table 1).

**Table 1:** Summary schedule of data collected

**Method of data collection**	**Screening**	**Baseline**	**Post-intervention**	**6-month follow-up**
ECBI & SCS*	**X**		**X**	**X**
Demographics		**X**		
FQ		**X**	**X**	**X**
PSQ			**X**	
Follow-up Questionnaire				**X**

ECBI, Eyberg Child Behavior Inventory; FQ, Family Questionnaire; PSQ, Parent Satisfaction Questionnaire; SCS, Social Competency Scale. *Screening is within 3 weeks of baseline data collection.

### Sample size

Previous research indicated that 80% of participants receiving HPS completed the IYP group [43,45]. Therefore, a total sample of 126 participants will be collected in order to achieve 50 participants in each treatment arm at post-treatment. This trial represents the first formal assessment of the HPS intervention and is being undertaken as a pilot study to assess the feasibility of a full randomised controlled trial in the wider clinical setting and to collect data to inform the power calculations for such a study. Thus, there is no formal power calculation for the proposed sample size of 126, but this represents a substantial and adequate number of participants representative of those likely to benefit from the intervention. Standard power calculations with 50 in each arm will have 80% power to detect an effect size of 0.57 between the control and experimental group (i.e., Cohen’s *d* = 0.57).

### Randomisation and sequence generation

On completion of baseline data collection, participants will be allocated an identification number and randomised to IYP plus HPS or to IYP alone. An independent statistician using a computer generated randomisation sequence generated prior to the enrolment of any participants will undertake the randomisation. Randomisation will be stratified on each IYP group so that each intake or source group will have approximately equal numbers allocated to each treatment. The randomisation sequence will allocate in a 1:1 ratio to the two treatments arranged in permuted blocks and will be stratified on age (under 5 years and over 5 years), sex, and ethnicity (Maori and Non-Maori). After a participant has met all inclusion criteria and signed informed consent they will be allocated the next available randomisation allocation.

### Allocation concealment

The randomisation list will not be available to any researchers directly involved in the assessment or screening of participants. The participant will only be allocated once all inclusion criteria are met. Following randomisation, participant allocation will be returned to the primary investigator who will inform participants of their allocation and arrange for HPS to begin in the treatment group.

### Blinding

Due to the nature of the study, it is not possible to have a completely blinded design. Participants will know which intervention they are receiving. IYP group leaders will also know who is in the treatment arm as their contribution is a part of the HPS intervention. The primary investigator leads the IYSS team and conducts the multidisciplinary team review and will therefore be aware of those participants in the treatment arm. However, the research assistant undertaking the assessments will be blind and remain blind to treatment allocation throughout the study. Participants will be asked not to reveal the intervention they are receiving to the research assistant. All participants will be given an identification number to ensure the researcher and all those involved in summarising and inputting the data are unaware of the treatment allocation.

### Statistical methods

Standard descriptive statistics will be used to report demographics, baseline status for outcome measures, and presentation features for the sample as a whole and by randomly allocated group. These will include means, medians, ranges, and standard deviations for metric measures, and frequencies and percentages for categorical measures.

The primary outcome measure, the change in the parent scores on the ECBI total problem score from pre- to post-intervention will be calculated for each individual and will be compared between randomised groups using ANOVA with randomised group and strata as fixed factors. Additional sensitivity analyses will be undertaken using an ANCOVA model and including the baseline level of the change score as a covariate.

The metric secondary outcome measures that assess change from pre- to post-intervention in SCS, and child behaviour, parenting practices, parent relationships and parental wellbeing as measured by the FQ, will also be compared between randomised groups using ANCOVA models with baseline levels as covariates and randomised group and strata as fixed factors.

The categorical outcomes at post-treatment including the percentage of parent scores on the ECBI and the SCS that are in the normal range at post-treatment and the percentage of participants with at least 80% engagement in IYP will be compared between randomised groups using χ^2^ tests.

As outlined above, the stratification factors will be included as factors in the ANCOVA models analysing the primary and secondary continuous outcomes and, depending on sample size, may also be included in a Mantel-Haenszel χ^2^ analysis of the post-treatment categorical outcomes.

The maintenance of post-treatment results for the primary and secondary outcomes at six months post-intervention will be compared between randomised groups using ANOVA. This analysis will explore change in the metric measures from immediately post-treatment to six months between the two randomised groups.

Additional exploratory analyses including correlation coefficients and further ANCOVA and logistic regression models may be used to identify the characteristics of subsets of participants who respond particularly well or poorly to the addition of HPS to IYP.

A two tailed α = 0.05 will be used for all statistical testing of the results of the above analyses and results will be summarised using 95% confidence intervals of the differences between randomised groups. Should any of the above metric outcome measures not meet requisite assumptions for parametric analyses after transformation, non-parametric tests, including the Mann-Whitney U-test, will be used for analyses.

All participants’ data will be included in the intention-to-treat analysis. Considerable efforts will be made to obtain post-treatment and follow-up data from all randomised participants even if they do not complete the treatments. Missing data will in the first instance be managed with a ‘last observation carried forward’ approach with additional sensitivity analyses undertaken using multiple imputation methods. The extent of compliance, including information on those who do not complete either HPS or IYP, will be captured and summarised. A per-protocol analysis, including only those who complete the treatments without protocol violations and have all relevant assessments at each time, will also be undertaken to identify whether compliance factors affect outcomes.

### Qualitative analysis

A small number of qualitative questions are included in the questionnaires to assess participants’ unique perspective and experience of the intervention. At baseline, open questions include reasons for referral to IYP and asking parents about their expectation of the intervention. Post-treatment questions explore the parents experience of the intervention they received (HPS or IYP alone) and what, if any, benefits they have gained. Follow-up questions focus on changes in child behaviour and parent–child relationships. Questions also focus on the parents’ experience of being part of the trial and any suggested improvements.

Responses will be coded using a general inductive approach described by Thomas [46]. All responses will be read systematically to identify meaningful units. These will be coded and then categorised into emerging themes. Any links or relationships between the themes will be established. The frequent, dominant, or significant themes will be identified, and will inform research findings. Participants’ responses to open ended questions are expected to give insight into the impact of child behaviour on the family, their expectations and hope for change, and their experience of the intervention, including unplanned or unanticipated effects. An independent coder will code 30% of transcripts to ensure reliability of coding. Any discrepancy in themes will be resolved by agreement between the two coders (Additional file 1).

## Discussion

There is considerable evidence for the efficacy of IYP for most families who are experiencing challenges with child behaviour. Research shows that up to two thirds of families who complete IYP have child behaviour rating scores in the normal range at post-treatment and this is maintained at follow-up [5,13]. For those families whose children do not make sufficient change during treatment, the risk of later poor outcomes is raised substantially. These families may respond to extra in-home support to encourage engagement in IYP, address barriers for making change, and support the implementation of effective parenting strategies. We anticipate that providing tailored in-home coaching to vulnerable families while they are attending IYP will result in more participants having post-treatment child scores in the normal range. A structured therapist guide has been developed to ensure the intervention is delivered with fidelity.

It is costly to provide intervention and treatment for conduct disorder and the cost increases with age and severity. If the trajectory of just a few young children can be changed early in the life of the child then it is more likely that the improvement will be maintained over time and this can provide a saving to health, education, and social justice.

This is the first formal evaluation of adding a structured home intervention (HPS) to the IYP group-based programme and is a feasibility study to inform the design and implementation of a larger definitive randomised controlled trial. It is hypothesised that HPS will improve outcomes in families with risk factors for non-response to treatment, encourage them to stay engaged in IYP, strengthen their adoption of effective parenting strategies, and improve outcomes for both the children and the families. If a significant effect size is found this would justify expansion and development of HPS. However, if the effect size is small it could be concluded that HPS does not have additional benefit over IYP alone for the sample identified for this trial. These findings could provide information to inform National Ministries on policy and resource allocation.

## Trial status

Recruitment commenced in March 2013. The final participants are expected to complete their 6-month follow-up assessment in December 2014.

## Abbreviations

CAMHS: Child and Adolescent Mental Health Services; ECBI: Eyberg Child Behavior Inventory; FQ: Family Questionnaire; HPS: Home Parent Support; IYP: Incredible Years Parent Programme; IYSS: Incredible Years Specialist Service; SCS: Social Competency Scale.

## Competing interests

The authors declare that they have no competing interests.

## Authors’ contributions

DL was responsible for the conception and design of the study with advice from DF and SM. DL wrote the initial draft of the protocol. CF advised on the statistical analysis and established the database. SM and DF reviewed subsequent drafts of the manuscript. DL is responsible for the implementation of this study. All authors approved the final manuscript.

## Supplementary Material

Additional file 1: Figure S1 Participant flow. Click here for file
