# Supplementary figures and images for: A pilot study to evaluate the efficacy of adding a structured home visiting intervention to improve outcomes for high-risk families attending the Incredible Years Parent Programme: study protocol for a randomised controlled trial
Source: Trials. 2014 Feb 25;15:66. doi: 10.1186/1745-6215-15-66 (PMC3938816; doi:10.1186/1745-6215-15-66)

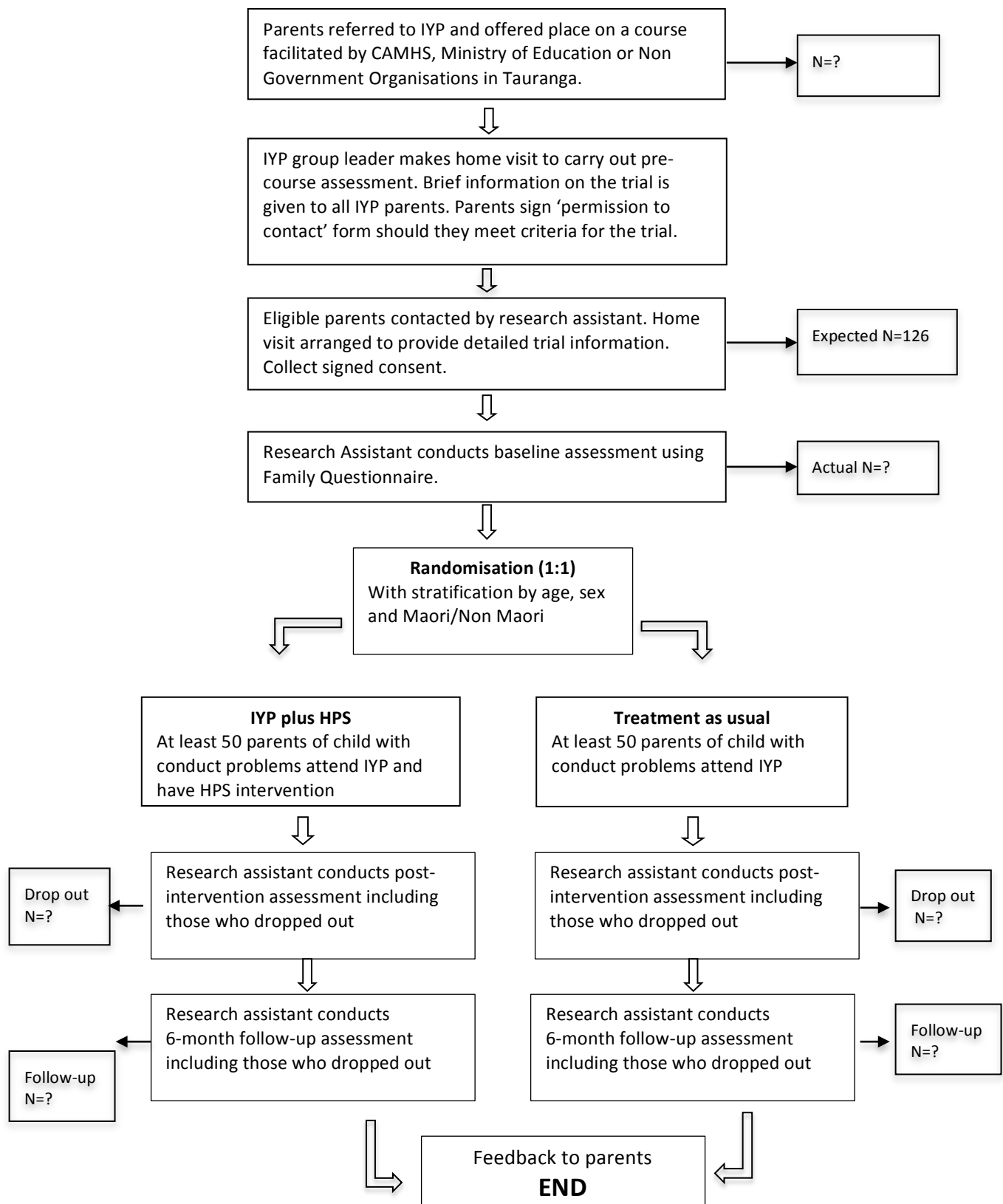

**FIGURE 1 Participant Flow**

Supplement: Additional file 1: Figure S1 — Participant flow. [file 1745-6215-15-66-S1.pdf]
